# Identifying Sources of Potential Bias When Using Online Survey Data to Explore Horse Training, Management, and Behaviour: A Systematic Literature Review

**DOI:** 10.3390/vetsci7030140

**Published:** 2020-09-22

**Authors:** Kate Fenner, Michelle Hyde, Angela Crean, Paul McGreevy

**Affiliations:** Sydney School of Veterinary Science, Faculty of Science, University of Sydney, Sydney, NSW 2006, Australia; michelle.hyde@sydney.edu.au (M.H.); angela.crean@sydney.edu.au (A.C.); paul.mcgreevy@sydney.edu.au (P.M.)

**Keywords:** owner-reported observations, horse behaviour, social license, survey bias, equine veterinary behaviour, domestic equine triad

## Abstract

Owner-reported behavioural observations form an essential part of the veterinarians’ diagnosis and treatment plan. The way we train and manage horses affects their behaviour and, in turn, their health and welfare. Current horse training and management practices are largely driven by traditional techniques and longstanding methodologies. These approaches generally lack an evidence base for evaluation purposes. The absence of evidence and evaluation contributes to the persistent use of risky practices and this, in turn, increases risk of potential harms for both horse and rider, and fuels questioning of the equine industry’s current social license to operate. Objective evidence is required to make training and management decisions based on demonstrable best practice. Large-scale experimental or intervention studies using horses are generally not practical because of the associated costs and logistics of gaining ethical approval. Small studies generally lack statistical power and are subject to the effects of many forms of bias that demand caution in the interpretation of any observed effects. An alternative to collecting large amounts of empirical data is the use of owner-reported observations via online survey. Horse owners are ideally placed to report on the domestic equine triad of training, management, and behaviour. The current article highlights three sources of potential bias in a systematic review of literature on large-scale online studies of horse owners’ observational reports that met the following selection criteria: English-language, published, peer-reviewed articles reporting on studies with over 1000 respondents and open access to the survey instrument. The online surveys were evaluated for three common forms of bias: recall, confirmation, and sampling bias. This review reveals that online surveys are useful for gathering data on the triad of horse training, management, and behaviour. However, current use of online surveys to collect data on equitation science (including horse training, management, and behaviour) could be improved by using a standardised and validated tool. Such a tool would facilitate comparisons among equine and equitation science studies, thus advancing our understanding of the impacts of training and management on horse behaviour. The authors of the current review suggest the use of a standardised behavioural and management assessment tool for horses. Such a tool would help define what constitutes normal behaviour within geographically disparate populations of horses, leading to improvements in rider safety and horse welfare.

## 1. Introduction

Domestic horses are generally very compliant animals [1], with an ethology that features exceptional behavioural flexibility and rapid habituation [2]. In the developed world, they are kept mainly as companion, leisure, or sporting animals rather than working animals and are handled by people with a diversity, and often paucity, of relevant skills [3]. Veterinary and husbandry interventions as simple as examinations or grooming can bring risks to human safety [4]. At the same time, traditional and contemporary training and management practices regularly compromise horse welfare across each of the Five Domains [5]. Shortfalls in horse welfare, and associated behavioural manifestations, can go unnoticed and lead to poor welfare outcomes for horses and increase hazards for riders, who are often unaware of the horse’s emotional state [4,6]. 

There is a triadic relationship among training, management, and behaviour. When flaws in training and management practices are left unresolved, a cycle of poor training and management can develop. For example, chasing a horse in a round pen will elicit a flight response which the horse is likely to associate with the round-pen environment and thus offer each time the round pen is encountered, thus jeopardising both horse welfare and handler safety [7]. These training flaws can lead to further deterioration in the horse’s behaviour and escalation in the use of force and coercion in training, followed by spiraling risks of flight responses, habituation to pressure cues and associated safety risks to riders and handlers. This cascade of ‘knock-on’ effects directly jeopardises horse welfare. Progress in understanding the influence of training and management practices on horse behaviour relies on the collection and analysis of large-scale data on each of these variables to establish a baseline and evaluate change. 

The concept of social license to operate (SLO) relates to community trust in a business, organisation, or entity operating within legal, cultural, social, and ethical norms, while striving for best practice. This is important because the traditional methods of animal handling, training, and management are increasingly being challenged [8,9]. Public attention has questioned whether industries, ranging from those using laboratory rodents to those using racing horses, maintain a SLO to use animals as they have done historically [10,11]. SLO is not a formal or written contract, rather society’s permission for an entity to continue to operate. With the rise of social media, which fuels the distribution of troubling images, the equestrian community risks losing its SLO unless it can be seen to be taking proactive, affirmative action to improve participant safety and horse welfare [12]. In recent years, a series of ridden horse welfare issues have been prominent across the media, risking their SLO. These include the use of Rolkur [13], whip use in racing [14], the use of restrictive nosebands in various sporting disciplines [15], and wastage within the breeding and racing industries [16]. Some of these (such as restrictive nosebands) reflect the perceived need to reduce unwelcome behaviour while others (such as wastage) directly result from unwelcome behaviour.

Horse training and management practices currently lack a robust evidence base, despite decades of research into equine and equitation science [7]. Many traditional practices are untested empirically and even studies that have tested training techniques may suffer from small numbers of practitioners [17]. To date, data collection tools for the use of horses in sport and leisure contexts have been disparate and largely unvalidated [18]. 

Horse owners are ideally suited to contribute data on the domestic equine triad of training, and management practices and their horses’ behaviour because they are generally very familiar with their animals [18]. Surveys have been used to collect data on horse behaviour, training, management, and health for over four decades [19] and online questionnaires for approximately twelve years [20]. Various disciplines, training and management practices have been investigated and owner-reported observations have already contributed considerably to our understanding of horse–human relationships [21,22,23]. However, these data have generally been collected with a narrow focus [24], small sample numbers [25], or a closed cohort of horses or riders, where inclusion was restricted to individuals meeting identified criteria [26], making it difficult to generalise their results across the equine population.

Nevertheless, online data collection has many benefits including cost effectiveness, the ability to recruit large numbers of respondents, and the ease and speed of global spread with the ability to target specific stakeholder groups. However, ease of use may have inadvertently led to online questionnaires lacking standardisation and validation, such that large numbers of rapidly produced surveys may have poor reliability, making results both difficult to evaluate individually and to compare to one another.

Historically, questionnaires for horse owners have collected both qualitative and quantitative data. The collection of qualitative data on horse owners’ and riders’ interpretation of behaviour has provided important insights into perceptions that potentially affect welfare. For example, research has shown that attitudes towards mares and geldings vary considerably, potentially putting welfare of mares at risk [27]. Furthermore, Visser and Van Wilk-Jensen [28] found significant differences among horse enthusiasts’ (trainers, riders and coaches) beliefs and practices around welfare issues. While horse-owner attitudes and beliefs merit scientific scrutiny, the collection of qualitative data on attitudes and beliefs is unlikely to further our understanding of what constitutes normal behaviour in horses or be useful when making geo-cultural comparisons [29].

Defining what constitutes normal behaviour in horses necessitates the collection of quantitative data. It is important when investigating relationships among training, management and behaviour, that horse owners are asked to report their observations rather than their opinions or interpretations of behaviours. Free-text commentaries on observed behaviour require two interpretation procedures to take place: first the owner interprets the behaviour they observe; and second, the researcher interprets their text response. A survey instrument that examines the frequency of behavioural observations reduces the effects that arise from errors in interpretation.

The horse–human dyad is more complicated than that of most other human–other animal interactions due to horses being ridden. Clearly, there is no equivalent horse–horse interaction to mounting and riding astride the horse. This complexity begins with mounting the horse for riding [30]. Prior to this stage of the horse’s education, much of the horse–human interaction—for example, stroking, grooming, and leading—can be plausibly aligned with conspecific interactions. All horses, especially those that are ridden or driven, require multifaceted management regimes and various levels of training. For example, an elite equine athlete might be housed in a stable full time, exercised using lunging, riding, and walking or even swimming. This same horse may have specialized tack and equipment, such as bits and headgear, which would not be used with horses in other disciplines. Alternatively, a horse may be housed in a large field and only interact with the owner once a month. However, in both cases, training and management, including interactions that handlers might not consider to be formal training, affect horse behaviour, which, in turn, reflects and contributes to the animal’s health and welfare status.

While online surveys are inexpensive, expedient, and able to reach vast numbers of potential respondents, we must address the inherent risk of bias with this method of data collection. All online surveys are likely exposed to both self-selection bias, where only individuals with internet access who choose to visit a website and decide to participate can respond [31], and non-response bias, where participants are unwilling or unable to answer questions [32]. A further three types of bias are more readily addressed, including recall bias (participants fail to remember past events accurately), confirmation bias (participants interpret information in a way that is consistent with their existing beliefs), and sampling bias (the survey respondent sample does not represent the population). Finally, our ability to disseminate study results has a direct impact on their uptake and, thus, usefulness. The current systematic literature review investigates questionnaires that have been used to obtain data from horse owners. Its objective was to examine the current use of online surveys to understand how and where large datasets are being collected and review their reliability, by assessing recall, confirmation, and sampling bias, in achieving stated aims. The chief goal was to identify best practice for large-scale data collection and dissemination on the domestic equine triad of horse training, management, and behaviour.

## 2. Materials and Methods

A systematic literature review aims to minimise bias by pre-specifying the eligibility criteria to address a specific research question. To maximise the chance that all relevant studies were captured in the current review, the initial search was as broad and simple as possible. A title, abstract and keywords search of three large databases (Web of Knowledge, Scopus, and PubMed) using the search terms ‘equine OR horse’ AND ‘survey OR question *’ AND ‘behav *’ was conducted on 15 January 2020. After removal of duplicates, 813 unique articles were identified for screening. Titles and abstracts were reviewed by two independent reviewers (KF and AC), and 444 articles excluded from further review because they did not have an equine focus. The remaining 181 articles were retained for full-text review and were examined against our specified inclusion criteria (Table 1).

Of the 181 articles identified for full-text review, 146 were removed due to being either a review article (*n* = 26) or because the surveys had fewer than 1000 respondents (*n* = 113). A further 11 articles were excluded because they were not written in English. Only English-language articles were included in this review because the research team lacked a reliable translation service and considered that, to appraise each instrument fully, such a service would be essential, given the nuances of describing behaviour and framing questions in different languages [33]. A further 13 studies were excluded as they were not open surveys. An open survey was defined as being available to any horse owner/caregiver and not restricted to a defined population, for example ‘wind-sucking’ horses. Of the 20 articles classified as open survey, seven were rejected because they were qualitative investigations examining horse owners’ beliefs and opinions, rather than observational reports about an individual horse’s behaviour, training, or management. An additional article [34] authored by the current research group and meeting our inclusion criteria was not identified by the online search but found during hand searching. The search was repeated on 1 July 2020 and two further articles [15,35], one of which was authored by the current research group, that were published after the initial literature search was conducted were also included, leaving a final set of 14 articles for full review and quality assessment.

Online surveys, as with other methods of data collection, are subject to various sources of bias [36]. When assessing reliability for our literature review, three potential sources of bias were considered as themes, and the included articles were given a subjective rating out of five on each theme (Table 2):

(1) Recall bias may arise when participants are required to remember past events, with the length of the recall period increasing the likelihood of this bias occurring. In our review, we considered the lag between the behaviours or management practices occurring and their being reported. Studies that asked participants to report on their horses’ behaviour or management in the ‘previous week’ [37,38,39,40,41,42,43] scored five out of five. In contrast, a study [44] that asked respondents to report on behaviours witnessed during a specific event a year previously scored two out of five (see Table 2).
(2) Confirmation bias may arise when a participant must rely on their preconceptions or beliefs to answer questions. We reviewed the survey items used and rated the studies according to their apparent objectivity. Studies that asked respondents only for simple observations [43,45], such as ‘does your horse wear a blanket?’ or ‘is your horse kept in a group?’, scored five out of five; whereas studies with questions that required considerable interpretation by the participant [37], such as ‘does the horse like to get its own way?’, scored three out of five (see Table 2).
(3) Sampling bias may arise if a particular demographic of survey participants has a higher or lower probability of being included than others, thus affecting how representative the findings might be of the general horse population. For example, the work of Hartmann et al. (2017) reported on winter housing in a cold climate and generated a low score for sampling bias as the sampled population was located in a country with a climate not relevant to the global horse population [45]. Conversely, a high score would be obtained if the survey investigated behaviours that were unlikely to be geographically influenced [42] (see Table 2).

The scoring system (Table 2) was developed by the authors to rank bias themes between studies. Recall bias is a measure of how far back participants were required to remember when answering questions. Confirmation bias explored each question on the survey and rated it on a 5-point scale as objective (frequency with which a behaviour was observed or management practice used, score 5) to subjective (requiring considerable interpretation from participants, score 1). Finally, sampling bias assessed the relevance of all items within the survey to the entire equine population.

## 3. Results

There were 14 studies meeting the inclusion criteria (Table 1). Data extracted from these studies included the behaviours and/or management practices investigated, the objectives of the studies and the conclusions drawn by the authors (Table 3). The included studies covered a wide range of behaviours and management techniques, horse breeds and ages and participant demographics. The scope of geographical coverage was somewhat limited, with the majority (8/14) of studies collecting data in the UK only. Many of the studies (6/14) came from a single UK-based research group. While all of the surveys were available online and open to the public, most (12/14) were advertised only in their own immediate geographical location and some (8/14) recruited participants solely from those geographical areas [United Kingdom (5), Scandinavia (2), Australia (1), and New Zealand (1)].

Bias scores, agreed by both assessors, for each of the three items were collated to give articles a total indicative score out of 15 (see Table 3).

## 4. Discussion

The considerable number of studies found, in the first sweep of the current search, and the breadth of topics covered are testament to the endeavours of equine and equestrian scientists. The selected studies incorporated a wide range of topics including training interventions, management practices, and ridden and in-hand (on the ground) behavioural problems, with large sample sizes, demonstrating the important contribution such surveys make to our understanding of core elements within the horse–human dyad. 

The search and selection criteria for the current review revealed 14 relevant, original research articles. Critical analysis of these articles confirmed the potential for three main sources of bias that reduced, at least in some part, the reliability of their results. Some of the identified limitations are more obstructive to the drawing of conclusions than others and are more readily overcome in future attempts to embrace best practice. For example, sample bias is somewhat easier to address in survey design and participant selection. Confirmation bias can be avoided by not requiring participants to interpret their horses’ behaviour or motivations for performing a behaviour. For example, rather than asking respondents whether their horse was ‘anxious’, simply asking them to report how frequently the horse performed a given behaviour, such as running along the fence, requires no interpretation and is thus less likely to reflect confirmation bias. 

The authors of the current review recognise and work within the described restrictions inherent in large-scale data collection themselves and, as such, acknowledge and applaud the efforts made by the designers of the studies discussed here. There is little doubt that these studies have significantly advanced knowledge in the field. However, our review also reveals that the quality of data collected via online surveys varies. This reflects the dearth of survey instruments that are standardised, validated, and longitudinal. Standardisation across instruments facilitates valid comparisons among populations of horses. The use of a survey instrument that has been subjected to inter- and intra-reporter reliability increases confidence in both the survey and its findings. 

The formulation of objective questions that simply ask participants to report on the frequency with which they observe behaviours should be a top priority for questionnaire designers and a deciding factor on the reliability of the resulting behavioural data. For example, in their survey of the management of horses during fireworks, Gronqvist et al. [44] acknowledged this limitation, but still chose to ask participants to rate ‘*how anxious their horses were*’ when exposed to fireworks. Perhaps if they had asked participants about the behaviours they observed, such as running the fence, vocalising and so on, their survey would have produced a more robust, and thus generalisable, result. In the same vein, attempting to ascribe anthropomorphic terminology such as personality traits to horses may not be useful. For example, Ross et al.’s [47] participants were required to include interpretation in their responses, e.g., to report whether their horse displayed *‘an anxious expression’* when headshaking.

Attempting to guess a horse’s motivations for behaving in a certain way can lead to erroneous assumptions and jeopardise welfare [48]. For example, Lloyd et al. [49] asked participants to assess their horses’ personality using a 7-point Likert scale with pre-defined adjectives such as *‘Motherly—Provides warm receptive secure base for others, is tender and caring’*, and concluded that, despite the answer requiring considerable participant interpretation, the use of the anthropomorphic terminology was more useful than an objective description of the animal’s behaviour. However, as respondents likely differ in their definition of ‘tender and caring’, perhaps the insertion of requests for objective behavioural observations might be a useful addition to such inquiries. In such cases, simply asking horse owners or caregivers to report on the behaviours observed (that may or may not have led them to conclude that the horse was ‘tender and caring’) would be useful information to collect.

As there is currently no validated, standardised tool available for exploring the domestic equine triad, researchers have had to start afresh each time a behavioural survey is undertaken, as demonstrated in our results. This results in a collection of dissimilar survey tools, making comparisons between studies impossible. A single questionnaire, tested for construct validity and reliability, would solve this problem while building a behavioural database that is greatly needed. With these baseline data in place, researchers could ask for additional information on topics of interest or interventions, providing validity and enabling comparison between populations.

Online survey tools can provide useful cross-sectional data but, when building an evidence base from which to establish a description of normal behaviour and investigate the effect of training and management regimes, it is important to source data at different time points. Longitudinal studies are required to investigate the consequences of training and management over time. By retesting the same population at regular intervals, using the same standardised and validated instrument, one can draw evidence-based conclusions on topics that affect rider safety and horse welfare. Unfortunately, none of the studies in the current review were longitudinal. While this may reflect the current nature of scientific endeavour in the equine and equitation science fields and the reality that these disciplines are essentially still in their infancy [50], the current authors suggest that the collection of longitudinal data should be seen as an opportunity to enhance the disciplines.

The current review reveals that data on the domestic equine triad of training, management, and behaviour for focal horses need to be collected concurrently, and repeated measurements need to be taken longitudinally to reveal how the elements of the triad interact. A longitudinal survey tool will allow collection of data that can be analysed to reveal how changes in training and management manifest as behaviour, and how these behavioural changes influence horse welfare. While not all survey objectives demand the collection of longitudinal data, our search results indicate that no large-scale longitudinal data on the domestic equine triad have been collated.

The collection of longitudinal data may also assist when considering patterns in responded data over time. Recall bias is largely a function of the specific timeframe participants are invited to report on [51]. Inaccuracies in recall are an important limitation when surveying participants in many areas of science, and are particularly notable in the field of nutrition [52]. Hockenhull and Creighton [38,39,40,41] managed this by instructing their participants to report on the previous week’s observations, whereas Gronqvist et al.’s [44] participants were asked to report on events that occurred a full twelve months previously. In contrast, Hartmann et al.’s [43,45] management practice surveys specified that respondents were to report on horses currently in their care, thus maximising the possibility of returning accurate results. By allowing respondents to report on horses that were no longer in the possession of the participants, either having been sold or deceased, Ross et al. [47] were, arguably, relying too heavily on owners’ recollections.

The quality of data collected is limited by the validity and reliability of the survey instrument applied. While the risks of bias, as discussed in this review, can be minimised by a carefully crafted questionnaire, this does not validate the instrument or ensure it collects reliable data among various respondents or over time. The current authors suggest the need for a three-part validation and reliability assessment process [53]. Construct validity can be assessed by asking respondents to give a general assessment of their horse and compare that to their results. This has been used to validate the Feline Behavior Assessment and Research Questionnaire (Fe-BARQ) [54], wherein respondents were asked “Are you currently experiencing any problems with this cat’s behavior or temperament?”, and answers were found to align with the results of the Fe-BARQ, providing some construct validity. Reliability testing requires two assessments, intra-rater and inter-rater reliability assessments. Intra-rater reliability can be assessed by the same respondent reporting on a horse twice over a given period. To show intra-rater reliability, results from both assessments must align. Finally, inter-rater reliability testing involves two respondents reporting on the same horse. Questionnaires containing objective questions, reporting on frequency of behaviours observed, should reveal significant alignment between observers. Inter-rater reliability testing will likely reveal, notably where there is a lack of correlation, those questionnaire items that have required respondents to interpret behaviour. It is recommended that future large-scale data collection enedeavours incorporate each of these validation and reliability testing protocols to assure the quality of findings and enhance the scientific knowledgebase. 

Dissemination of results may be greatly assisted by two elements that should be incorporated into any future questionnaires. The first is making questionnaires available to a global population. This aim can be greatly advanced by translating the survey into several different languages and thus avoid an Anglo-centrically focused database. Translations, to overcome language barriers, need to be conducted by content experts to ensure precision and accuracy [55]. Social media and extensive networking tools that are currently available make distributing online questionnaires easier than ever before. While one survey tool will never answer every question researchers hope to ask, a standardised and eventually validated method can provide scientists with baseline behavioural data from which to contextualise their investigation.

Administering a standardised behavioural instrument across different experimental settings will allow results to be generalisable, contribute to our understanding of what constitutes normal behaviour in horses and reveal relationships among variables not previously investigated. Baseline behavioural indices allow researchers to understand the influence of the age and breed of horses on behaviour. Further, once that a baseline has been established, one can begin to discern the effect of management and training practices. Perhaps Hartmann’s et al. [43,45] studies on management practices, while geographically limited to Nordic countries with typically cool climates, may have benefitted from such baseline behavioural indices. 

The dissemination of results from citizen science activities, such as pooling of horse-owner observations, is critical when feeding back to participants [56]. Dissemination is greatly assisted by open access publication of study results. It is interesting to note that of the 14 papers reviewed here, only three, [15,35,44], were published in an open access journal. This is concerning because horse welfare relies on the dissemination of clear and accurate research results, particularly to horse owners and caregivers who are responsible for the largest group of domestic horses, the pleasure riding community [57]. It is interesting to consider that a possible source of publication bias arises due to the higher costs of open access publication restricting this type of publication to large institutions or well-funded research groups. We recognise that many authors do not have the resources to fund open access.

## 5. Conclusions

Collecting data on horse training, management and behaviour using owner-reported observations is a simple, efficient, and inexpensive procedure. The use of online surveys vastly increases the potential reach and participation rate of studies, greatly increasing the ability to obtain robust datasets. Current large-scale online surveys have greatly strengthened our understanding of the use of equipment, how management practices are adapted in various geographical regions, and how training and management interact with behaviour. The data obtained from owners could be optimised by ensuring that behavioural questions do not need to be interpreted by owners, thus reducing confirmation bias. The prospects for integrating the collection of behavioural data into a global citizen science project are strong. A standardised and repeatedly accessible questionnaire could potentially change the way numerous stakeholder groups benchmark and understand horse behaviour and provide a baseline description of what constitutes normal behaviour across all geographic locations. 

## Figures and Tables

**Table 1:** Inclusion and exclusion criteria used for the literature review investigating the use of online surveys for data collection on horse training, management, and behaviour.

Criterion	Inclusion	Exclusion
Targeted respondents	Equine owner/caregiver/rider, any age or gender	Studies restricting participation by limiting availability to specific cohorts
Core demographic data	Breeds, disciplines, sex of horse, country, behaviour, training, management	
Questionnaire	Online, unrestricted url access, English-language, peer-reviewed original research articles	Non-English publications, review articles
Respondent numbers	Studies describing 1000 individual horses or more	Studies describing fewer than 1000 individual horses
Data collected	Quantitative/observational data reported on individual horses	Studies investigating horse owners’ beliefs or opinions

**Table 2:** Scoring system used for three themes that relate to potential bias in surveys.

	Score				
Theme	1	2	3	4	5
Recall bias	>1 year	up to 1 year	3 to 6 months	<1 and >3 months	Current or up to 1 month
Confirmation bias	Completely subjective interpretation	More subjective than observational	Both subjective and objective	More observational than subjective	Completely observational
Sampling bias	Relevant to very specific equine populations	Extremely limited to climatic or geographical relevance	Some climatic or geographical limitations	Partial climatic or geographic relevance	Relevant to all equine populations

**Table 3:** Included reviews (Ref.) characteristics and reliability assessment [(recall bias (RB), confirmation bias (CB), and sampling bias (SB) with total reliability score (Total)] of studies using online surveys of >1000 participants to quantify horse behaviour.

Ref.	Behaviours Investigated	Objectives (*n* = Number of Respondents)	Outcomes/Conclusions	RB	CB	SB	Total/15
[42]	Oral investigation, licking, searching, and biting/nipping	To investigate whether giving treats by hand, or the use of clicker training, was associated with oral behaviours including searching for food, licking and biting (*n* = 1067)	No significant associations between hand feeding and nipping/biting clothing were found. The use of clicker training was not associated with unwelcome oral behaviours.	5	4	5	14
[35]	Training, management, and behaviour	To explore behavioural differences between ridden mares and geldings (*n* = 1233)	No significant differences were found in ridden behaviour but mares were more likely to move away when being caught and geldings were more likely to chew on lead ropes when tied and rugs.	3	5	5	13
[43]	Human behaviour (housing practices)	How horse owners in these four countries keep their horses and their understanding of the importance of the horse’s need for social contact. How management practices relate to horse breed and owner opinions (*n* = 3229)	Most horses were kept in groups for at least part of the 24 h period and those never grouped were primarily competition horses or stallions. Horses are usually kept in similar age and sex groups.	5	5	3	13
[39]	Stable-related and handling problems	To discover the prevalence of stable-related behavioural problems when handling horses (*n* = 1850)	A high prevalence of handling and stable-related problems was found, indicating that restricted turnout might have welfare costs.	5	4	4	13
[40]	15 ridden behavioural problems	To investigate the use of equipment and training practices and the prevalence of 15 behavioural problems in UK leisure horses (*n* = 1326)	The high prevalence of reported behavioural problems (91%) in ridden horses suggests a risk to rider safety and horse welfare.	5	4	4	13
[41]	Prefeeding behaviour (aggression, frustration, and stereotypies)	To generate data on diet and owners’ perceptions of the use of supplements, feeding practices and the prevalence of behavioural problems encountered prior to feeding in UK leisure horses (*n* = 1324)	Current feeding and management regimes may compromise horse welfare.	5	4	4	13
[38]	Stereotypic behaviour and problem behaviour	To assess the risk factors associated with routine management practices on behavioural problems in UK leisure riding horses (*n* = 1226)	Different groups of behavioural problems were associated with different management risk factors. These leisure horse findings are similar to those found in performance horses.	5	4	4	13
[37]	Personality traits—excitability, anxiousness, dominance, and protection	To explore potential breed differences in horse personalities using a trait theory approach (*n* = 1223)	Horse breeds were found to differ in their personality types, and this finding was found to align with the traditional views on breed differences.	5	3	5	13
[45]	Human behaviour (blanketing/clipping)	The extent of blanketing and clipping horses in Sweden and Norway and owners’ knowledge of the literature surrounding their use and heat loss (*n* = 6197)	While blanketing and clipping are very common practices in Sweden and Norway, owners understanding of the horse’s thermoregulation and the literature behind these practices is limited.	5	5	2	12
[46]	Behavioural problems (stable related, handling and prefeeding)	The use of statistical techniques to identify associations and clusters of behaviours that could reflect an underlying welfare issue (*n* = 1681)	Behaviour clusters were found and may represent an underlying welfare issue, but this is not recognised by owners or traditional horsemanship methods. This leads to major welfare concerns. Once these patterns have been recognised by collecting data and analysing it, this information needs to be passed on to owners to enable them to take early action and improve horse welfare.	4	4	4	12
[34]	Human behaviour (equipment use)	To establish benchmark data on the prevalence of the use of an apparatus to apply aversive stimuli to ridden horses and ponies. The use of bits, nosebands, whips and spurs were investigated (*n* = 1101)	Crank nosebands in dressage and curb bits in Western disciplines could pose a risk to welfare. Whips and crops are used more in dressage and spurs by Western riders.	3	3	5	11
[47]	Headshaking	To discover the prevalence of headshaking observed within the last year in horses in the UK and its relations to sex and breed (*n* = 1014)	The estimated prevalence of headshaking was 4.6% and 75.4% of respondents had attempted at least one form of treatment trial. Headshaking was not related to sex or breed.	3	3	5	11
[15]	Human behaviour (equipment choice, perceived effectiveness and complications)	The distribution of noseband use, owner-reported reasons for use, perceived effectiveness, design choices, tightness monitoring and detrimental consequences (*n* = 2332)	A plain cavesson was the most commonly used noseband (46.6%), and 18.6% of users reported at least one physical or behaviour complication from noseband use.	3	2	5	10
[44]	Behaviours—running, vocalisation, and breaking through fences	The effects of fireworks on horses, horse responses and management interventions employed (*n* = 1111)	A total of 39% of horses were rated as ‘anxious’ or ‘very anxious’, resulting in running (82%) and breaking through fences (35%). While 77% moved their horses as a management strategy, this was ineffective 30% of the time.	2	3	2	7
